# Genome-Wide Analysis of DNA Methylation Signatures Linking Prenatal Exposure to the Chinese Great Famine and Blood Lipids in Late Adulthood: The Genomic Research of the Chinese Famine (GRECF) Study

**DOI:** 10.3390/nu17193147

**Published:** 2025-10-02

**Authors:** Huan Wang, Luqi Shen, Tingting Liu, Ruiyuan Zhang, Zhenghe Wang, Jingkai Wei, Ye Shen, Jinzhen Guo, Toni Miles, Changwei Li, Zhiyong Zou

**Affiliations:** 1 Institute of Child and Adolescent Health, School of Public Health, Peking University, Beijing 100191, China; 2211110224@bjmu.edu.cn; 2 Westlake Laboratory of Life Sciences and Biomedicine, Hangzhou 310024, China; shenluqi@westlake.edu.cn; 3 School of Nursing, Florida State University, Tallahassee, FL 32306, USA; tliu5@fsu.edu; 4 Department of Epidemiology, O’Donnell School of Public Health, University of Texas Southwestern Medical Center, Dallas, TX 75390, USA; ruiyuan.zhang@utsouthwestern.edu; 5 Department of Epidemiology, School of Public Health, Southern Medical University, Guangzhou 510515, China; wzh116086@smu.edu.cn; 6 Department of Family and Community Medicine, McGovern Medical School, University of Texas Health Science Center at Houston, Houston, TX 77030, USA; 7 Department of Epidemiology and Biostatistics, College of Public Health, University of Georgia, Health Sciences Campus, 101 Buck Road, Athens, GA 30602, USA; yeshen@uga.edu (Y.S.); tonimile@uga.edu (T.M.); 8 Chinese Institutes for Medical Research, Beijing 100069, China; guojinzhen@cimrbj.ac.cn

**Keywords:** DNA methylation, prenatal, Chinese Great Famine, blood lipids, nutrition

## Abstract

Background/Objectives: Prenatal exposure to famine can lead to lasting health effects through changes in DNA methylation. This study aims to evaluate the impact of prenatal exposure to the Chinses Great Famine (1959–1961) on human epigenome and the subsequent influence on blood lipids. Methods: We conducted an epigenome-wide association study (EWAS) of peripheral blood-based DNA methylation and prenatal exposure to the Chinese Great Famine as well as blood lipids among eight participants exposed to famine and eight sex-matched participants (born ≤ 3 years after the famine). Genome-wide DNA methylation sites were profiled using the Illumina EPIC BeadChip, which covers 850K methylation positions. Results: After EWAS analyses, seven probes in genes *C8orf31*, *ELAVL1*, *U6*, *GBA2*, *SHOX2*, *SLC1A4*, and *NPHP4* reached *p* < 1 × 10^−5^. Of these, famine exposure was associated with decreased methylation levels of a GBA2 exonic probe cg08258661 (*p* = 4.9 × 10^−6^). After false discovery rate (*FDR*) correction, pathway enrichment analyses for genes harboring nominally significant (*p* < 0.05) probes identified 44 significant pathways (*q* < 0.05), and 5 pathways were related to lipid metabolism. After *FDR* correction in each pathway, probes cg02622866 (5’UTR of *ATF2*, *p* = 1.09 × 10^−3^), cg07316730 (body of *GRB2*, *p* = 1.32 × 10^−3^), and cg01105385 (body of *PIK3R1*, *p* = 1.94 × 10^−3^) in the PI2K-Akt signaling pathway were associated with blood LDL-C (*q* ≤ 0.04); probes cg09180702 (3’UTR of *PIGQ*, *p* = 9.21 × 10^−5^, and *q* = 0.04) and cg01421548 (body of *HS3ST4*, *p* = 5.23 × 10^−5^, and *q* = 0.01) in the metabolism pathway were associated with blood LDL-C and HDL-C, respectively; In addition, probe cg08460387 (5’UTR of *MAN1C1*, *p* = 1.09 × 10^−4^, and *q* = 0.02) in the vesicle-mediated transport pathway was associated with log-transformed blood triglycerides. Conclusions: Through an epigenetic study of the Chinese Great Famine, we identified six novel genes involved in lipid metabolism.

## 1. Introduction

Malnutrition during the prenatal period is associated with a series of health problems indicated by high mortality, morbidity, and mental disability [1,2]. The fetal origins hypothesis developed by Barker and colleagues is a core explanation for the observed associations. The hypothesis posits that an insufficient supply of nutrition and oxygen to the fetus and the resulting growth retardation cause abnormal structural development and malfunction of the human body later in life through “biological programming,” which in turn increases risks for obesity, coronary heart disease, type 2 diabetes, stroke, and hypertension in adulthood [3,4,5,6]. Changes in DNA methylation (DNAm) may be the “biological programming”. In the fetal stage, genome-wide demethylation occurs thereby driving gene expression and embryo development [7]. Adverse experiences during gestation may affect the demethylation process [8,9,10], resulting in changes that persist throughout life and exert a risk for chronic diseases in later life [10]. Early life exposure to severe nutrition deprivation, such as famine, provides an unparalleled opportunity to exam the fetal origins hypothesis.

In our previous studies, we discovered that 11.6% of the middle-aged and older Chinese adults with an immediate family member starved to death during the Chinese Great Famine (1959–1961) [11] and prenatal exposure to the Chinese Great Famine for up to three years was associated with an increased risk of dyslipidemia [12]. The Dutch famine studies have reported that participants with exposure to famine for up to six months during pregnancy had lower methylation levels in late adulthood at the *IGF2* and *INSIGF* loci and higher methylation levels at the *IL10*, *LEP*, *ABCA1*, *GNASAS*, and *MEG3* loci, compared to their sex-matched siblings without famine exposure [9,10]. Additionally, Tobi and colleagues demonstrated that DNAm at genes *TXNIP*, *LOC100132354*, *PNPO*, *LRRC8D*, *ABCG1*, and *SYNGR1* mediated associations of famine with the body mass index and triglycerides [13]. Another epigenome-wide association study (EWAS) on early life exposure to the Chinese famine found that fetal exposure to famine was predominantly linked with decreased DNA methylation levels, with the most enriched biological pathways related with neurodevelopment, neuropsychological disorders, and metabolism [14]. Despite new evidence on the epigenetic patterns of DNA methylation related the Chinese famine, its subsequent impacts on adult metabolic health remain to be further investigated.

Given that the Chinese Great Famine lasted much longer with more extreme severity, especially in the Anhui Province, and post-famine food supply was not fully recovered until twenty years later in the 1980s, an epigenetic study of the Chinese Great Famine may help to identify novel genomic loci underlying blood lipid traits in late adulthood. Therefore, we conducted an EWAS based on the Chinese Great Famine to identify novel genomic loci in which methylation changes were associated with prenatal famine exposure. We also aimed to evaluate associations of famine-related DNAm changes with blood lipids in late adulthood.

## 2. Materials and Methods

### 2.1. Study Participants

The Genomic Research of the Chinese Famine (GRECF) study was designed to investigate the impact of early life exposure to the Chinese Great Famine on metabolic disorders in late adulthood. Participants were recruited from two neighboring provinces, Anhui and Jiangxi, in China through a multi-stage, clustered, random sampling. First, we randomly selected one urban region and one rural region in each province. Second, three counties in each urban region and three villages in each rural region were randomly selected. Third, thirty to thirty-five subjects in each county or village born within three years of the Chinese Great Famine were randomly selected based on birthdate, which was obtained from the National Resident Registration System. Finally, a total of 790 subjects who consented to participate and completed the anthropometric assessments and survey questionnaires were enrolled into the GRECF.

The two provinces were chosen as study sites because of their large difference in famine severities and similarity in geographic location, ethnic composition, lifestyle, and dietary habits (Supplemental Table S1). Anhui province was the most severely affected by famine across the entire nation. According to our previous studies, 35.1% of the middle-aged and older adults in Anhui province reported having immediate family members starve to death [11]. Furthermore, based on the 1990 Census of Chinese Population, the average population size of adults who were born during the three years of the famine (1959–1961) was 63% smaller compared to those born in the three years immediately before or after the famine [15,16,17,18]. Jiangxi is a neighboring province of Anhui but has a much smaller cohort size shrinkage index (0.35) and lower prevalence of severe famine experience (6.8%). The current study was approved by the Institutional Review Board at the Peking University Health Science Center (Approval number: IRB00001052-15057; Approval date: 24 September 2015). All study participants have signed informed consent forms to participate in the study.

### 2.2. Prenatal Famine Assessment

The current study was conducted among 16 GRECF participants born in Anhui province. Specifically, four male and four female participants born in Anhui province during the famine were randomly selected as the group with prenatal famine exposure (born between 1 October 1959 and 30 September 1961). Then, eight sex- and location of residence-matched GRECF participants born shortly after the famine (<3 years; born between 1 October 1962 and 30 September 1964) in Anhui province were selected as the control group.

### 2.3. DNA Methylation Measurements

Genome-wide DNAm sites in peripheral blood were profiled using the Illumina EPIC BeadChip, which covers 850K methylation positions. DNAm levels were calculated as β-values, which represent the percentage of DNA that is methylated at the interrogated CpG sites and ranges from 0 to 1. Normal-exponential convolution using the out-of-ban probes method was used to correct background noise, normalize dye, and scale type I and II probes [19], making high quality β-values for probes comparable [19]. Heterogeneity of cell types were corrected based on DNAm levels previously identified in purified blood cell lines [20]. Quality control removed probes based on any of the following criteria: (1) probes with an intensity level at or close to the background intensity (*p* > 0.01); (2) probes that failed to hybridize as a minimum of three beads; (3) probes that located in a DNA sequence containing known SNPs with minor allele frequency >5%; and (4) probes that mapped to multiple locations [21].

### 2.4. Blood Lipid Measurements

Participants were asked to fast for at least eight hours before examination. A four milliliters tube of the fasting blood sample was drawn for each participant by trained nurses between August 2015 and May 2016. A unique barcode was generated for each participant and attached to the blood tubes. After collection, the fresh venous blood samples were transported at 4 °C to the central laboratory at the Peking University Health Science Center, where whole blood samples were separated into plasma and blood cells and stored at −80 °C in a deep freezer. Total cholesterol (TC), high-density lipoprotein cholesterol (HDL-C), low-density lipoprotein cholesterol (LDL-C), and triglycerides were quantified from plasma using the enzymatic colorimetric tests in a Roche cobas 4000 analyzer (Roche Diagnostics, Indianapolis, IN, USA) at the central laboratory.

### 2.5. Anthropometric Measurements

Height, weight, and waist circumference were measured using standardized protocols by trained staff. Waist circumference was measured to the nearest 0.1 cm at the level of the umbilicus, with two repeated measurements taken for each participant and the average value used in the analysis. Body mass index (BMI) was calculated as weight in kilograms divided by height in meters squared (kg/m^2^). Blood pressure was measured on the right arm using a mercury sphygmomanometer (model XJ11D, Shanghai Medical Instruments Co. Ltd., Shanghai, China). Supplemental Table S2 presents the anthropometric indicators and blood lipid profiles of the 16 GRECF participants.

### 2.6. Statistical Analyses

For characteristics of participants, independent sample *t*-test was used to compare differences in continuous variables between famine-exposed group and non-famine-exposed group, and Chi-square test was used to compare differences in categorical variables across groups.

Beta values for each probe were compared between individuals with and without prenatal famine exposure using the empirical Bayes statistics implemented in Limma software [22,23]. Probes with a raw *p* value < 0.05 were mapped to genes using Kobas [24], followed by enrichment analyses for pathways in KEGG, Reactome, and PANTHER [25]. Methylation sites in genes of significant pathways involved in lipid metabolism were tested for associations with blood lipids using multivariate linear regression adjusting for sex, monthly family total income, and individual education attainment level. We also performed genome-wide analyses to identify all CpG sites associated with blood lipids adjusting for sex, monthly family total income, and individual education attainment level. Statistical significance was defined as a false discovery rate (FDR)-adjusted q value < 0.05 for pathways and CpG sites. For probes in lipid-related pathways, FDR correction was conducted separately in each pathway. Statistical analyses were conducted with software R version 4.4.2 (http://www.R-project.org). All *p* values were two-sided and *p* or FDR-corrected *q* values < 0.05 were considered as being statistically significant.

## 3. Results

### 3.1. EWAS Results for Prenatal Famine Exposure

A total of 48055 CpG positions showed nominal associations (*p* < 0.05) with prenatal famine exposure, of which, 137 CpG sites reached *p* < 1 × 10^−4^, the significance level that was used in a previous EWAS of the Dutch famine (Supplemental Table S3). Furthermore, seven CpG positions reached *p* < 1 × 10^−5^ (Table 1), specifically, prenatal famine exposure was associated with increased methylation levels in cg10360725 (5’UTR of *C8orf31*), cg04359527 (TSS of *ELAVL1*), and cg11736566 (5’UTR of *U6*), and decreased methylation levels in cg08258661 (exon of *GBA2*), cg09542210 (body of *SHOX2*), cg02085294 (5’UTR of *SLC1A4*), and cg06173758 (body of *NPHP4*).

### 3.2. Replication of Previous Reported DNAm Changes Associated with Famine

We compared our results with previously reported EWAS for famine exposure (Supplemental Tables S4 and S5). We successfully replicated 11 out of 13 genes identified in the Dutch famine epigenetic study. In a separate Chinese famine EWAS based on the Qingdao population, we replicated five CpGs, whereas only one CpG (cg05083630, *p* = 0.036) remained nominally significant in our EWAS results.

### 3.3. Enrichment Analysis

After FDR correction, genes harboring nominally significant probes were significantly enriched in 44 pathways of the KEGG, Reactome, or PANTHER (Table 2), of which 20 pathways are broadly related to metabolism, growth and development, and/or energy expenditure (Table 2), 6 pathways are involved in immune function, 4 pathways are involved in the nervous system, and the remaining pathways are related to cancer risk, homeostasis, and general functions. Of the significant pathways, five are related to lipids metabolism: R-HSA-1430728 (metabolism: *p* = 6.76 × 10^−11^, *q* = 2.88 × 10^−7^), R-HSA-392499 (metabolism of proteins: *p* = 3.14 × 10^−7^, *q* = 3.82 × 10^−4^), R-HSA-5653656 (vesicle-mediated transport: *p* = 3.65 × 10^−6^, *q* = 0.02), R-HSA-556833 (metabolism of lipids and lipoproteins: *p* = 1.34 × 10^−4^, *q* = 0.03), and hsa04151 (PI3K-Akt signaling pathway: *p* = 2.41 × 10^−4^, *q* = 0.04).

### 3.4. Associations of DNAm with Blood Lipids

We performed analyses of CpG sites mapped to genes within those five lipid-related pathways for associations with blood lipids (Table 3). After FDR correction in each pathway, CpG sites in genes *ATF2* (cg02622866), *GRB2* (cg07316730), and *PIK3R1* (cg01105385) of the *PI3K-Akt* signaling pathway (ID: hsa04151) and *PIGQ* (cg09180702) of the metabolism pathway (ID: R-HSA-1430728) were significantly associated with blood LDL-C. One CpG site, cg01421548 in *HS3ST4* of the metabolism pathway (ID: R-HSA-1430728) was associated with blood HDL-C. Probe cg08460387 in the 5’UTR region of the *MAN1C1* gene of the vesicle-mediated transport pathway (ID: R-HSA-5653656) was significantly associated with logarithmically transformed blood triglycerides. Additionally, two CpG sites, cg02344276 (5’UTR of *TNS1*: *β* = −8.9, *p* = 1.99 × 10^−7^, and *q* = 0.04) and cg06758928 (intergenic: *β* = −6.0, *p* = 2.47 × 10^−7^, and *q* = 0.04) were not in the lipid-related pathways but were significantly associated with logarithmically transformed blood triglycerides after a genome-wide FDR correction. The two probes were not associated with prenatal famine exposure, either.

## 4. Discussion

Through genome-wide analyses, a total of 48055 CpG sites showed nominal associations with prenatal famine exposure. The seven CpG sites had *p* values less than 1 × 10^−5^. Prenatal famine exposure was associated with higher methylation levels in three of the CpG sites and lower methylation levels in the other four sites. Genes with CpG sites nominally associated with prenatal famine exposure were significantly enriched in 44 pathways, of which 20 pathways are broadly related to metabolism, growth and development, and/or energy expenditure, and 5 pathways are related to lipid metabolism. Analyses to CpG sites within the genes of the five pathways related to lipid metabolism revealed that CpG sites in *ATF2*, *GRB2*, *PIK3R1*, and *PIGQ* were associated with blood LDL-C, one CpG site in *HS3ST4* was associated with blood HDL-C, and one CpG site in *MAN1C1* was associated with blood triglycerides. Furthermore, CpG site cg02344276 in the *TNS1* gene and intergenic site cg06758928 were not associated with famine exposure but were significantly associated with lower blood triglycerides. These findings highlight the impact of prenatal malnutrition on epigenomic changes in humans and help delineate the mechanisms underlying lipid regulation.

The top seven CpG sites associated with prenatal famine exposure were located in genes *C8orf31*, *U6*, *SLC1A4*, *ELAVL1*, *GBA2*, *NPHP4*, and *SHOX2*. Of the seven genes, *C8orf31*, *SLC1A4*, *GBA2*, and *SHOX2* are functionally relevant to metabolism and/or development. Notably, the cg08258661 site, situated in the first exon of the *GBA2* gene, encodes glucosylceramidase beta 2, an enzyme involved in carbohydrate transport and metabolism [26]. Although cg08258661 was not associated with blood lipid traits in the present study, a nearby CpG site in the *GBA2* (cg11046700) was associated with lower triglycerides (β = −22.6, *p* = 0.005). Of note, according to the first human imprint control region (ICR) compendium established by Jima and colleagues [27], ICR_14 is located within the *NPHP4* gene, suggesting that *NPHP4* is a likely target of direct imprinting regulation. Furthermore, 20 of the 44 significantly enriched pathways are related to metabolism, development, and/or energy expenditure. This highlights that prenatal famine exposure impacts metabolic disorders through epigenetic changes to the human genome.

Imprinted genes and metastable epialleles are two key epigenetically labile gene subsets of the genome that act as critical mediators linking early environmental exposures to adult disease susceptibility [28]. Metastable epialleles are particularly sensitive to periconceptional nutritional status, generating systemic and persistent interindividual epigenetic variations [29]. Although our study did not validate previously reported metastable epialleles associated with prenatal nutrition [30], prenatal malnutrition may still exert lasting effects through both stochastic alterations at metastable epialleles and perturbations at imprinted loci. This dual vulnerability highlights a plausible mechanism by which early nutritional adversity programs long-term susceptibility to lipid dysregulation. Future studies that systematically investigate candidate ICRs and metastable epialleles in famine-exposed cohorts could therefore provide stronger mechanistic evidence linking developmental programming, epigenetic regulation, and adult cardiometabolic health.

Five of the significantly enriched pathways were related to lipid metabolism. Further investigations of CpG sites of those pathways revealed that six genes, *ATF2*, *GRB2*, *PIK3R1*, *PIGQ*, *HS3ST4*, and *MAN1C1* had CpG sites significantly associated with LDL cholesterol, HDL cholesterol, and triglycerides. The *ATF2* gene encodes activating transcription factor 2 and regulates lipid and glucose metabolism through inducting PPARr coactivator and phosphoenolpyruvate-carboxykinase expression in animals [31,32]. *ATF2* is also involved in insulin resistance, β-cell function, and complications of type 2 diabetes [33]. The *GRB2* gene encodes the growth factor receptor-bound protein 2, which binds the epidermal growth factor receptor and is involved in the activation of Erk MAP kinase, an important pathway regulating lipid synthesis [34,35,36]. The *PIK3R1* encodes phosphoinositide-3-kinase regulatory subunit 1, an enzyme that plays a pivotal role in the action of insulin, and mutation in this gene has been associated with insulin resistance. C-terminal mutation in the *PIK3R1* among humans can cause severe insulin resistance with well-preserved liver fat and lipid profiles [37]. Furthermore, Jamshidi and colleagues demonstrated that variants in the *PIK3R1* gene were associated with apoB and LDL cholesterol [38]. The current study provided further evidence that methylation of this gene is associated with LDL cholesterol. The *PIGQ* gene encodes phosphatidylinositol glycan anchor biosynthesis class Q and is involved in glycosylphosphatidylinositol (GPI) anchor biosynthesis [39]. The GPI anchor protein primarily locates on the cell surface and pathways involved in lipid metabolism may affect their intracellular trafficking [40]. The *HS3ST4* gene encodes heparan sulfate-glucosamine 3-sulfotransferase 4, an enzyme that helps to generate heparan sulfate [41]. The heparan sulfate is a receptor for herpes simplex virus type 1 (HSV-1) and plays an important role in HSV-1 pathogenesis [42]. Interestingly, HSV-1 was observed to induce de novo phospholipid synthesis [43]. Furthermore, the *HS3ST4* gene was associated with obesity-related traits [44]. The *MAN1C1* gene encodes mannosidase alpha class 1C member 1 [45], while the role of this gene in lipid metabolism is still unclear. Taken together, these findings highlighted the long-term impact of prenatal famine exposure on lipid metabolism in late adulthood.

Pathway enrichment analyses also revealed six significant pathways involved in immune function and four relevant to the nervous system, indicating that prenatal famine exposure may impact both the immune and nervous systems. These findings may help explain the observed associations of prenatal famine exposure with cognitive impairment among Chinese adults [46,47,48]; however, a direct relationship between prenatal famine exposure and autoimmune disease has not been explored. Interestingly, the increased risk for autoimmune disease has been observed in patients with eating disorders [49]. Future studies in this area are warranted to further investigate these potential links.

CpG site cg02344276 in the *TNS1* gene and intergenic site cg06758928 were not associated with famine exposure but were significantly associated with lower triglycerides. The *TNS1* gene encodes tensin-1, a protein involved in signal transduction [50]. The functional relevance of this gene in lipid metabolism is unclear. However, this gene was associated with coronary artery disease [51] and body height [52]. The cg06758928 is close to *SUCLA2*. The *SUCLA2* gene encodes the succinate-CoA ligase ADP-forming beta subunit, a mitochondrial matrix enzyme that plays an important role in metabolism. Genome-wide association studies have identified that variants in this gene were associated with visceral adipose in women [53].

As one of the genome-wide epigenetic studies of the Chinese Great Famine, our study has important strengths. Compared to a previous famine-related EWAS in Qingdao [14], our study participants came from Anhui, which was the most severely affected province by the Chinese famine, providing a stronger potential to identify novel epigenetic changes due to extreme malnutrition during the early life stage. Moreover, our study further investigated the link between blood lipids in adulthood and prenatal famine-related DNAm levels, which will shed light on the role of DNAm in lipid metabolic health from a lifelong perspective and provide innovative evidence on Barker’s fetus programming hypothesis. In contrast to EWAS from foreign famines, such as the Dutch famine and Leningrad siege famine, the Chinese Great Famine served as a better quasi-experimental setting to explore the effect of prenatal famine on DNAm changes and further blood lipids in adulthood. First, during the 3-year Chinese Great Famine, about 11.6% of the middle-aged or older individuals reported immediate death of a family member due to starvation [11]. Therefore, epigenetic changes in Chinese people who experienced famine may be more prominent because of the extreme severity and long period of famine, enabling the identification of important CpG sites with even a small sample size. In addition, our findings are consistent with reports that environmental exposure increases the probability of methylation over the life span [54]. Second, the Chinese Great Famine is an important historical event because it was not complicated by war and was associated with a strictly governed society prohibiting migration. So, it is exempted from some potentially confounding factors, such as psychological stress associated with war and violent experiences. Last, most previously studied famines happened in wealthy societies where residents suffered from temporary starvation and then recovered with abundant food supplies right after the famine. In contrast, even after the 1959-61 famine, the food supply was limited for a couple decades in China until the market economy reforms in the 1980s.

However, our study has several limitations. First, due to the small sample size, our analysis was likely underpowered to detect subtle but biologically important epigenetic changes. We may have missed novel methylation sites resulting from prenatal famine exposure. Furthermore, the generalizability of our findings might be constrained. Future large-scale genome-wide epigenetic studies on the Chinese Great Famine are needed to identify more epigenetic changes linking prenatal famine exposure and metabolic disorders. Second, our DNAm data were derived from whole blood, which may not accurately reflect tissue-specific DNAm patterns. However, previous studies have shown that DNAm patterns in whole blood are often correlated with those in other tissues and can serve as reliable biomarkers for systemic metabolic traits, including blood lipid profiles. Third, our study did not explore the potential intergenerational effects of prenatal famine exposure. Previous studies on this topic have yielded inconsistent findings, with some reporting evidence of transgenerational effects [55] while others failed to detect similar transmissions to the next generation [56]. Given the possibility that famine exposure in the parental (F0) generation could influence not only survivors themselves but also their offspring (F1) and even grandchildren (F2), future research is warranted to systematically investigate these intergenerational effects, thereby advancing our understanding of the long-term and multigenerational consequences of famine exposure. Finally, although we excluded the confounding effects of several socioeconomic factors, such as family income and individual education level, unmeasured confounding, such as concentrations of free fatty acids and phospholipids, which are closely linked to blood lipids, may still have influenced our results.

## 5. Conclusions

In conclusion, the current genome-wide epigenetic study identified 44 pathways influenced by prenatal famine exposure. Twenty of the pathways are broadly related to metabolism, development, and/or energy expenditure. Five pathways are directly or indirectly associated with lipid metabolism. Analyses of the CpG sites in genes of the lipid-related pathways identified six novel CpG sites associated with blood lipids. Meanwhile, two CpG sites not associated with prenatal famine exposure were negatively associated with blood triglycerides.

## Figures and Tables

**Table 1:** Seven CpG sites associated with prenatal famine exposure with the lowest *p* values.

Probe ID	Chr: POS (Build 37)	Site Function	Nearest Genes	Beta Values		*p* Values
				Exposed Group	Matched Control	
cg10360725	8:144139316	5’UTR	C8orf31	0.36	0.95	2.7 × 10^−7^
cg04359527	19:7980718	TSS	ELAVL1	0.16	0.25	4.7 × 10^−6^
cg08258661	9:35748665	Exon	*GBA2*	0.03	0.02	4.9 × 10^−6^
cg09542210	3:157821536	Body	*SHOX2*	0.07	0.04	5.4 × 10^−6^
cg11736566	4:57155953	5’UTR	U6	0.87	0.92	7.5 × 10^−6^
cg02085294	2:65220148	5’UTR	*SLC1A4*	0.90	0.82	7.9 × 10^−6^
cg06173758	1:5957461	Body	*NPHP4*	0.94	0.91	9.5 × 10^−6^

**Table 2:** Significant pathways enriched with genes that methylation was at least nominally associated with prenatal famine exposure.

Terms	Pathway ID ^1^	*p* Values	*q* Values
Pathways in cancer	hsa05200	7.94 × 10^−6^	3.98 × 10^−3^
Hemostasis	R-HSA-109582	5.00 × 10^−5^	1.94 × 10^−2^
General function			
Gene expression	R-HSA-74160	1.19 × 10^−11^	1.01 × 10^−7^
Signal transduction	R-HSA-162582	1.51 × 10^−6^	1.31 × 10^−3^
Disease	R-HSA-1643685	1.54 × 10^−6^	1.31 × 10^−3^
Generic transcription pathway	R-HSA-212436	2.39 × 10^−6^	1.85 × 10^−3^
Transmembrane transport of small molecules	R-HSA-382551	2.60 × 10^−6^	1.85 × 10^−3^
Vesicle-mediated transport	**R-HSA-5653656**	3.65 × 10^−5^	1.56 × 10^−2^
Post-translational protein modification	R-HSA-597592	7.44 × 10^−5^	2.35 × 10^−2^
Extracellular matrix organization	R-HSA-1474244	1.04 × 10^−4^	3.07 × 10^−2^
Downstream signal transduction	R-HSA-186763	1.06 × 10^−4^	3.07 × 10^−2^
Membrane Trafficking	R-HSA-199991	1.19 × 10^−4^	3.26 × 10^−2^
Cell cycle	R-HSA-1640170	2.77 × 10^−4^	4.69 × 10^−2^
Growth and development			
Developmental biology	R-HSA-1266738	7.91 × 10^−8^	1.69 × 10^−4^
Signaling by PDGF	R-HSA-186797	3.62 × 10^−5^	1.56 × 10^−2^
Signaling by EGFR	R-HSA-177929	6.92 × 10^−5^	2.35 × 10^−2^
Signaling by FGFR2	R-HSA-5654738	1.08 × 10^−4^	3.07 × 10^−2^
Signaling by FGFR3	R-HSA-5654741	1.23 × 10^−4^	3.26 × 10^−2^
Signaling by FGFR	R-HSA-190236	1.29 × 10^−4^	3.26 × 10^−2^
Signaling by FGFR4	R-HSA-5654743	1.32 × 10^−4^	3.26 × 10^−2^
Downstream signaling of activated FGFR4	R-HSA-5654716	1.63 × 10^−4^	3.42 × 10^−2^
Downstream signaling of activated FGFR2	R-HSA-5654696	1.63 × 10^−4^	3.42 × 10^−2^
Downstream signaling of activated FGFR3	R-HSA-5654708	1.63 × 10^−4^	3.42 × 10^−2^
Signaling by FGFR1	R-HSA-5654736	1.71 × 10^−4^	3.42 × 10^−2^
Downstream signaling of activated FGFR1	R-HSA-5654687	1.97 × 10^−4^	3.74 × 10^−2^
Wnt signaling pathway	P00057	2.08 × 10^−4^	3.86 × 10^−2^
Signaling by Wnt	R-HSA-195721	2.36 × 10^−4^	4.20 × 10^−2^
PI3K-Akt signaling pathway	hsa04151	2.41 × 10^−4^	4.20 × 10^−2^
Signaling by SCF-KIT	R-HSA-1433557	2.80 × 10^−4^	4.69 × 10^−2^
Immune function			
Immune system	R-HSA-168256	1.54 × 10^−7^	2.62 × 10^−4^
Adaptive immune system	R-HSA-1280218	4.73 × 10^−6^	2.69 × 10^−3^
Fc epsilon receptor (FCERI) signaling	R-HSA-2454202	7.24 × 10^−5^	2.35 × 10^−2^
DAP12 interactions	R-HSA-2172127	1.74 × 10^−4^	3.42 × 10^−2^
Innate immune system	R-HSA-168249	1.74 × 10^−4^	3.42 × 10^−2^
DAP12 signaling	R-HSA-2424491	2.17 × 10^−4^	3.94 × 10^−2^
Metabolism			
Metabolism	**R-HSA-1430728**	6.76 × 10^−11^	2.88 × 10^−7^
Metabolism of proteins	**R-HSA-392499**	3.14 × 10^−7^	3.82 × 10^−4^
Metabolic pathways	**hsa01100**	2.92 × 10^−6^	1.91 × 10^−3^
Metabolism of lipids and lipoproteins	**R-HSA-556833**	1.34 × 10^−4^	3.26 × 10^−2^
Nervous system			
Axon guidance	R-HSA-422475	7.89 × 10^−7^	8.41 × 10^−4^
Neuronal system	R-HSA-112316	3.49 × 10^−6^	2.12 × 10^−3^
Signaling by NGF	R-HSA-166520	6.07 × 10^−6^	3.23 × 10^−3^
NGF signaling via TRKA from the plasma membrane	R-HSA-187037	3.34 × 10^−5^	1.56 × 10^−2^

^1^ Bolded are pathways related to lipids metabolism.

**Table 3:** Significant CpG sites in genes of the lipid metabolism-related pathways associated with blood lipids.

Probe ID	Pathway	Position	Nearest Gene	Function	*β* (*SE*)	*p* Values	*q* Values ^1^
High-density lipoprotein cholesterol							
cg01421548	>R-HSA-1430728	>16:25705355	> *HS3ST4*	>Body	>5.7 (1.0)	>5.23 × 10^−5^	>0.04
Low-density lipoprotein cholesterol							
cg02622866	hsa04151	2:176032527	*ATF2*	5’UTR	−140.7 (34.4)	1.09 × 10^−3^	0.03
cg01105385	hsa04151	5:67584222	*PIK3R1*	Body	65.0 (17.1)	1.94 × 10^−3^	0.03
cg07316730	hsa04151	17:73316721	*GRB2*	Body	41.7 (10.4)	1.32 × 10^−3^	0.03
>cg09180702	>R-HSA-1430728	>16:633722	> *PIGQ*	>3’UTR	>−6.9 (1.3)	>9.21 × 10^−5^	>0.04
Log triglycerides							
cg08460387	R-HSA-5653656	1:26000291	*MAN1C1*	Body	51.6 (9.7)	1.09 × 10^−4^	0.02

^1^ False discovery rate-corrected *q* values in each pathway.

## Data Availability

All the raw data presented in this study can be provided upon request by the corresponding authors.

## Supplementary Materials

The following supporting information can be downloaded at: https://www.mdpi.com/article/10.3390/nu17193147/s1, Table S1: Prevalence of severe famine exposure and cohort size shrinkage index in all provinces in China; Table S2: Participant characteristics by fetal famine exposure status; Table S3: CpG sites reaching *p* values < 1 × 10^−4^; Table S4: Replication analysis results for genes identified in the methylation studies of the Dutch famine; and Table S5: Replication analysis results for genes identified in the methylation studies of the Great Chinese Famine in Qingdao, China.

## Abbreviations

The following abbreviations are used in this manuscript:

Chr	chromosome
CI	confidence intervals
CpG	5’-C-phosphate-G-3’
EWAS	epigenome-wide association study
FDR	false discovery rate
GRECF	the genomic research of the Chinese famine
HDL-C	high-density lipoprotein cholesterol
HSV-1	herpes simplex virus type 1
ICR	imprint control region
LDL-C	low-density lipoprotein cholesterol
POS	position
SE	standard error
TC	total cholesterol
TSS	transcription start site
UTR	untranslated region
