# Supplementary material for: Genome-Wide Analysis of DNA Methylation Signatures Linking Prenatal Exposure to the Chinese Great Famine and Blood Lipids in Late Adulthood: The Genomic Research of the Chinese Famine (GRECF) Study
Source: Nutrients. 2025 Oct 2;17(19):3147. doi: 10.3390/nu17193147 (PMC12526373; doi:10.3390/nu17193147)
Supplement: Supplementary file 1 [file nutrients-17-03147-s001.zip › nutrients-3876080-supplementary.pdf]

Table S1. Prevalence of severe famine exposure and cohort size shrinkage index in all provinces in China

Province	CSSI	Prevalence (95% *CI*)	
		Severe famine[1]	No starvation
Anhui	0.63	35.1 (28.5,41.7)	45.9 (40.8,51.1)
Sichuan	0.61	28.3 (23.6,33.1)	55.4 (51.3,59.5)
Chongqing	0.54	24.0 (14.3,33.6)	57.6 (47.2,67.9)
Hunan	0.54	15.1 (10.6,19.7)	64.1 (58.6,69.6)
Guizhou	0.52	21.4 (4.7,38.0)	58.5 (42.8,74.2)
Qinghai	0.52	13.5 (12.0,14.9)	67.7 (43.0,92.3)
Henan	0.47	12.8 (9.8,15.7)	61.2 (57.4,64.9)
Guangxi	0.46	7.6 (4.3,10.9)	66.4 (61.6,71.2)
Gansu	0.45	21.2 (15.6,26.9)	55.4 (43.6,67.2)
Jiangsu	0.42	9.7 (6.6,12.7)	65.8 (61.9,69.7)
Hubei	0.42	8.5 (4.6,12.4)	71.0 (65.4,76.6)
Shandong	0.40	9.5 (5.1,13.9)	65.2 (58.7,71.7)
Yunnan	0.38	8.1 (5.7,10.5)	71.3 (67.3,75.3)
Fujian	0.37	7.7 (5.0,10.4)	63.1 (58.9,67.2)
Zhejiang	0.35	1.8 (0.4,3.1)	57.6 (49.9,65.3)
Jiangxi	0.35	6.8 (4.9,8.8)	70.0 (64.0,76.0)
Guangdong	0.35	12.6 (0.0,31.5)	61.7 (49.5,74.0)
Hebei	0.34	6.7 (3.6,9.8)	73.9 (70.8,77.0)
Tianjin	0.33	6.2 (0.4,12.1)	72.4 (54.8,89.9)
Liaoning	0.32	6.0 (3.8,8.2)	73.8 (67.2,80.4)
Shanxi	0.26	5.4 (0.9,9.9)	71.8 (65.3,78.3)
Shaanxi	0.25	3.6 (1.8,5.3)	70.9 (66.0,75.8)
Jilin	0.23	5.3 (0.0,10.8)	73.5 (62.2,84.8)
Beijing	0.22	7.4 (0.0,18.8)	43.9 (30.7,57.1)
Shanghai	0.22	1.9 (0.0,11.4)	48.6 (14.1,83.1)
Xinjiang	0.22	7.3 (0.0,27.9)	57.4 (0.0,100.0)
Inner Mongolia	0.20	4.3 (1.8,6.9)	66.9 (57.9,76.0)
Heilongjiang	0.19	2.7 (0.0,5.9)	65.0 (55.0,75.1)

Abbreviations: CI, confidence interval. CSSI, cohort size shrinkage index;

[1]Severe famine was defined as having immediate family members starve to death.

Table S2. Participant characteristics by fetal famine exposure status

Characteristics	Non-exposed (n=8)	Exposed (n=8)	t/χ^2 values	P values
Date of birth	10/01/1962–09/30/1964	10/01/1959–09/30/1961	–	–
Age, year	52.1±0.8	55.5±0.8	13.98	<0.001
Male, *n* (%)	4 (50.0)	4 (50.0)	0.00	1.000
Height, cm	162.3±8.0	158.8±9.0	0.84	0.417
Weight, kg	65.1±12.2	65.8±18.1	-0.08	0.935
WC, cm	86.0±12.5	84.9±17.4	0.14	0.891
BMI, kg/m^2	24.5±2.6	25.9±5.9	-0.59	0.567
SBP, mmHg	137.1±20.9	142.9±23.9	-0.51	0.616
DBP, mmHg	86.2±7.4	91.8±13.5	-1.01	0.334
FPG, mmol/L	6.4±2.2	5.5±0.4	1.14	0.290
TC, mmol/L	5.1±0.9	5.6±0.9	-1.10	0.288
TG[1], mmol/L	1.2 (0.8, 1.9)	1.2 (1.1, 1.4)	30.00	0.875
HDL-C, mmol/L	3.1±0.8	3.6±0.6	-1.29	0.217
LDL-C, mmol/L	1.5±0.4	1.6±0.5	-0.58	0.574
Meat intake ≥ once/day, *n* (%)	5 (62.5%)	5 (62.5%)	0.00	1.000
Fruit intake ≥ once/day, *n* (%)	3 (37.5%)	2 (25.0%)	0.00	1.000
Vegetable intake ≥ once/day, *n* (%)	8 (100.0%)	8 (100.0%)	0.00	1.000
Milk intake ≥ once/day, *n* (%)	2 (25.0%)	2 (25.0%)	0.00	1.000
Abdominal obesity, *n* (%)	3 (37.5%)	4 (50.0%)	0.00	1.000
Hypertension, *n* (%)	5 (62.5%)	8 (100.0%)	1.64	0.200
High FPG, *n* (%)	4 (50.0%)	0 (0%)	3.00	0.083
High TG, *n* (%)	3 (37.5%)	2 (25.0%)	0.00	1.000
Low HDL-C, *n* (%)	1 (12.5%)	1 (12.5%)	0.00	1.000
MS[2], *n* (%)	3 (37.5%)	3 (37.5%)	0.00	1.000
CVD[3], *n* (%)	1 (12.5%)	0 (0%)	0.00	1.000

Data are mean ± standard deviation or *n* (%). BMI: body mass index; CVD: cardiovascular disease; DBP: diastolic blood pressure; FPG: fasting plasmas glucose; HDL-C: high-density lipoprotein cholesterol; LDL-C: low-density lipoprotein cholesterol; MS: metabolic syndrome; SBP: systolic blood pressure; TC: total cholesterol; TG: triglyceride; WC: waist circumference; Independent sample t-test was used to compare differences in continuous variables between famine exposed group and non-famine exposed group, and successive corrected Chi-square test was used to compare differences in categorical variables across groups.

[1.] TG is presented as median (Q1, Q3) and Wilcoxon rank sum test is conducted for group difference in TG between famine and non-famine groups;

[2] Based on the 2013 Chinese Diabetes Society criteria, metabolic syndrome is defined when an individual meets any three or more of the following five components: 1) Abdominal obesity: Waist circumference ≥90 cm (men) or ≥85 cm (women); 2) Hyperglycemia: FPG ≥6.1 mmol/L or 2hPG ≥7.8 mmol/L and/or previously diagnosed diabetes; 3) Hypertension: BP ≥130/85 mmHg and/or previously diagnosed hypertension; 4) Hypertriglyceridemia: TG ≥1.70 mmol/L; 5) Low HDL-C: HDL-C <1.04 mmol/L;

[3.] CVD was defined as a self-reported physician diagnosis of stroke or cardiac diseases, including myocardial infarction, coronary heart disease, angina, or congestive heart failure.

Table S3. CpG sites reaching *P* values < 1E-04

CpG ID	CHR	MAPINFO	Exposed	Controls	Difference	*P*
cg10360725	8	144139316	0.36	0.95	-0.60	2.69E-07
cg04359527	19	7980718	0.16	0.25	-0.08	4.65E-06
cg08258661	9	35748665	0.03	0.02	0.01	4.87E-06
cg09542210	3	157821536	0.07	0.04	0.03	5.35E-06
cg11736566	4	57155953	0.87	0.92	-0.05	7.46E-06
cg02085294	2	65220148	0.90	0.82	0.08	7.94E-06
cg06173758	1	5957461	0.94	0.91	0.03	9.50E-06
cg05371438	12	184234	0.88	0.94	-0.06	1.02E-05
cg11309696	2	191183734	0.10	0.06	0.03	1.03E-05
cg08865534	1	95271674	0.07	0.13	-0.05	1.04E-05
cg11251178	5	75789532	0.93	0.89	0.03	1.15E-05
cg10499883	11	111076314	0.84	0.90	-0.06	1.26E-05
cg03910790	16	85376764	0.92	0.95	-0.03	1.36E-05
cg10158054	4	41662748	0.90	0.93	-0.03	1.46E-05
cg09727097	10	86297194	0.15	0.21	-0.06	1.51E-05
cg10380328	4	188953126	0.13	0.08	0.05	1.53E-05
cg10786870	19	44616116	0.88	0.82	0.06	1.59E-05
cg10948797	17	1957607	0.04	0.07	-0.03	1.61E-05
cg10553515	7	5013490	0.04	0.05	-0.02	1.63E-05
cg01520892	8	126970088	0.79	0.70	0.09	1.74E-05
cg07250080	6	32822278	0.07	0.15	-0.07	1.81E-05
cg10905330	1	213839708	0.89	0.80	0.09	1.84E-05
cg09684106	11	55640291	0.64	0.50	0.14	2.11E-05
cg05658414	6	10927915	0.88	0.83	0.05	2.13E-05
cg00594167	5	131772039	0.77	0.86	-0.09	2.14E-05
cg10781263	13	48507944	0.05	0.08	-0.04	2.35E-05
cg09704056	1	98516154	0.45	0.67	-0.22	2.51E-05
cg04938204	2	84529843	0.88	0.92	-0.04	2.86E-05
cg02113605	10	52162583	0.85	0.79	0.06	3.03E-05
cg09327219	10	114899819	0.96	0.98	-0.01	3.10E-05
cg02714465	6	14384133	0.96	0.94	0.02	3.14E-05
cg10501096	7	150424886	0.94	0.91	0.03	3.16E-05
cg01741372	11	783889	0.57	0.87	-0.30	3.22E-05
cg08239610	18	7958788	0.82	0.88	-0.07	3.22E-05
cg08548396	6	30095549	0.08	0.05	0.03	3.29E-05
cg09378441	7	36429182	0.04	0.07	-0.03	3.53E-05
cg04478931	4	86396739	0.20	0.12	0.08	3.56E-05
cg00160911	14	70041888	0.55	0.64	-0.09	3.69E-05
cg09059369	6	82574762	0.87	0.91	-0.05	3.93E-05
cg04413674	15	74494241	0.91	0.94	-0.03	3.98E-05

cg06333726	5	118691422	0.07	0.13	-0.05	4.01E-05
cg03740162	9	1050680	0.07	0.04	0.03	4.03E-05
cg08871016	21	45079232	0.10	0.16	-0.06	4.10E-05
cg04165683	2	109066028	0.03	0.04	-0.02	4.44E-05
cg11885173	10	29679151	0.60	0.69	-0.09	4.55E-05
cg02345908	10	1814106	0.91	0.85	0.06	4.58E-05
cg07721779	20	32891238	0.06	0.08	-0.03	4.61E-05
cg09877215	7	748801	0.93	0.90	0.03	4.63E-05
cg11590213	5	66331682	0.91	0.94	-0.03	4.65E-05
cg00931877	10	13672646	0.95	0.97	-0.02	4.71E-05
cg08579326	3	173271729	0.81	0.87	-0.07	4.76E-05
cg07614501	3	137493800	0.89	0.93	-0.04	4.80E-05
cg04187236	3	132378994	0.12	0.16	-0.04	4.82E-05
cg05174147	3	194554568	0.88	0.92	-0.04	4.97E-05
cg11756138	10	129216679	0.93	0.95	-0.02	5.02E-05
cg10977619	7	46673147	0.86	0.90	-0.05	5.11E-05
cg01277372	3	169378764	0.21	0.15	0.07	5.11E-05
cg06757436	1	229644978	0.93	0.96	-0.03	5.23E-05
cg00031303	3	195681400	0.18	0.09	0.08	5.25E-05
cg02929044	5	60561651	0.97	0.98	-0.01	5.44E-05
cg03957845	3	42307694	0.26	0.35	-0.09	5.59E-05
cg00977344	7	47816349	0.95	0.97	-0.02	5.63E-05
cg02699337	16	29312066	0.97	0.95	0.01	5.66E-05
cg04146151	16	2155961	0.82	0.91	-0.09	5.81E-05
cg10460130	2	242625978	0.06	0.10	-0.04	5.87E-05
cg01094121	6	28793540	0.33	0.22	0.10	5.87E-05
cg09739442	17	7387543	0.06	0.10	-0.04	5.91E-05
cg01208184	1	201744066	0.86	0.90	-0.04	5.96E-05
cg02083989	12	65153459	0.10	0.20	-0.09	6.27E-05
cg09674234	10	65225927	0.10	0.22	-0.12	6.36E-05
cg07335376	8	55047225	0.92	0.89	0.03	6.42E-05
cg08411924	10	118956130	0.86	0.90	-0.04	6.43E-05
cg09653046	4	129730880	0.04	0.05	-0.02	6.44E-05
cg09255968	10	60787807	0.91	0.97	-0.06	6.79E-05
cg03938711	12	121024974	0.88	0.92	-0.04	6.82E-05
cg03341758	20	34330234	0.04	0.07	-0.02	6.88E-05
cg03031660	17	73257791	0.04	0.06	-0.02	6.92E-05
cg03847895	12	56652945	0.45	0.62	-0.17	7.00E-05
cg03405092	7	79082017	0.08	0.05	0.03	7.01E-05
cg00828721	14	37121260	0.60	0.75	-0.16	7.07E-05
cg05611723	2	157179350	0.28	0.19	0.09	7.09E-05
cg02860602	12	103356060	0.15	0.10	0.05	7.10E-05
cg05915866	16	73090838	0.34	0.47	-0.13	7.16E-05

cg09269007	3	164914812	0.15	0.11	0.05	7.22E-05
cg00943756	2	130866915	0.81	0.70	0.11	7.23E-05
cg09856241	12	51442062	0.05	0.09	-0.03	7.26E-05
cg03010694	11	114423273	0.77	0.83	-0.07	7.37E-05
cg10846936	17	78151814	0.68	0.57	0.11	7.49E-05
cg04542603	9	71305158	0.87	0.91	-0.04	7.54E-05
cg09560911	6	47276890	0.04	0.02	0.01	7.57E-05
cg08920761	4	74563996	0.79	0.86	-0.08	7.70E-05
cg05310760	2	178500282	0.91	0.94	-0.03	7.79E-05
cg08802984	2	218141948	0.89	0.63	0.27	7.87E-05
cg11591030	3	105626744	0.21	0.13	0.08	7.88E-05
cg00075853	1	206213643	0.94	0.96	-0.02	7.88E-05
cg08656504	4	104119605	0.03	0.05	-0.01	7.94E-05
cg11209279	17	7146254	0.03	0.04	-0.01	7.96E-05
cg00922073	13	111364588	0.04	0.07	-0.02	7.99E-05
cg05712271	12	130396377	0.07	0.11	-0.04	8.01E-05
cg11731031	14	78265516	0.86	0.81	0.05	8.03E-05
cg07242860	5	140739522	0.08	0.13	-0.05	8.04E-05
cg03827386	3	15540665	0.93	0.90	0.03	8.10E-05
cg03439805	12	109124743	0.08	0.05	0.03	8.15E-05
cg09264396	2	88969320	0.21	0.32	-0.10	8.15E-05
cg03575556	14	21215971	0.89	0.92	-0.04	8.17E-05
cg08973061	15	98836506	0.05	0.08	-0.03	8.24E-05
cg02154186	8	26371353	0.32	0.24	0.08	8.28E-05
cg07077507	14	89290791	0.04	0.07	-0.02	8.34E-05
cg09372755	1	43919584	0.09	0.16	-0.07	8.36E-05
cg08972160	15	74486948	0.90	0.93	-0.03	8.46E-05
cg02690350	17	27620209	0.04	0.06	-0.02	8.47E-05
cg01546084	1	59394530	0.94	0.96	-0.02	8.49E-05
cg02956801	5	125758625	0.42	0.55	-0.13	8.54E-05
cg10930290	2	219840349	0.63	0.75	-0.12	8.63E-05
cg09517540	20	47231625	0.91	0.79	0.12	8.64E-05
cg08351474	20	4666916	0.05	0.10	-0.05	8.68E-05
cg08967200	11	28129733	0.05	0.08	-0.03	8.87E-05
cg09971499	10	76631573	0.22	0.16	0.07	8.91E-05
cg08418079	11	11448117	0.79	0.84	-0.05	9.11E-05
cg10950591	14	20820418	0.96	0.97	-0.02	9.21E-05
cg02515965	6	156885358	0.29	0.47	-0.18	9.25E-05
cg02473327	2	150080315	0.89	0.92	-0.03	9.31E-05
cg08871608	19	40729274	0.06	0.13	-0.07	9.33E-05
cg08627996	12	98973173	0.94	0.96	-0.02	9.42E-05
cg05922501	11	3636101	0.09	0.05	0.03	9.48E-05
cg10532290	10	119133434	0.04	0.05	-0.02	9.67E-05

cg06772063	16	54150765	0.85	0.89	-0.04	9.70E-05
cg08800613	2	1595498	0.82	0.67	0.15	9.76E-05
cg09137423	10	51827642	0.03	0.04	-0.01	9.82E-05
cg08862737	8	33461323	0.81	0.88	-0.06	9.84E-05
cg05494709	12	8396767	0.24	0.13	0.11	9.87E-05
cg10379687	20	44175957	0.81	0.87	-0.06	9.90E-05
cg10064248	10	101118946	0.87	0.91	-0.04	9.95E-05
cg01015739	3	100673290	0.88	0.92	-0.03	9.98E-05
cg01067849	6	2765587	0.04	0.08	-0.04	1.00E-04
cg05557255	11	2422385	0.05	0.07	-0.02	1.00E-04

Table S4. Replication analysis results for genes identified in the methylation studies of the Dutch famine

CpG ID	CHR	Position	Genes	Region	Exposed	Non-Exposed	Difference	*P*
cg02901679	1	206945999	IL10	TSS200	0.47	0.57	-0.10	0.005
cg05738152	2	287354	FAM150B	Body	0.24	0.16	0.08	0.003
cg05136031	7	127880567	LEP	TSS1500	0.47	0.55	-0.08	0.005
cg10389730	8	38585901	TACC1	1stExon	0.14	0.11	0.03	0.045
cg07396903	8	38600452	TACC1	5'UTR	0.67	0.63	0.03	0.040
cg11570784	8	38614280	TACC1	5'UTR	0.25	0.21	0.04	0.041
cg02565702	8	38614515	TACC1	5'UTR	0.06	0.04	0.01	0.014
cg11071960	8	38614832	TACC1	5'UTR	0.07	0.06	0.01	0.030
cg09560979	8	38615235	TACC1	5'UTR	0.06	0.04	0.01	0.004
cg08786644	9	107594238	ABCA1	Body	0.82	0.88	-0.05	0.002
cg10775893	9	107690540	ABCA1	TSS200	0.04	0.03	0.01	0.021
cg02719427	11	2151725	IGF2	3'UTR	0.96	0.96	-0.01	0.027
cg02045936	11	2152577	IGF2	3'UTR	0.95	0.94	0.01	0.049
cg11717189	11	2154132	IGF2	3'UTR	0.36	0.30	0.06	0.038
cg05384664	11	2154934	IGF2	Body	0.71	0.64	0.07	0.024
cg05452899	11	2161133	IGF2	Body	0.18	0.13	0.05	0.003
cg09694722	11	2161318	IGF2	Body	0.11	0.09	0.02	0.046
cg05444816	11	2162406	IGF2	TSS200	0.11	0.09	0.02	0.027
cg04112019	11	2165136	IGF2	5'UTR	0.05	0.04	0.01	0.040
cg05834354	11	3116865	OSBPL5	Body	0.93	0.92	0.01	0.034
cg07873128	11	3121400	OSBPL5	Body	0.92	0.88	0.03	0.014
cg05431723	11	3121590	OSBPL5	Body	0.86	0.80	0.06	0.009
cg09319649	11	3148917	OSBPL5	Body	0.92	0.89	0.02	0.036
cg08731067	11	3164631	OSBPL5	5'UTR	0.97	0.96	0.01	0.019
cg10803034	11	3178188	OSBPL5	5'UTR	0.82	0.76	0.05	0.030
cg11678461	11	3181822	OSBPL5	5'UTR	0.12	0.10	0.02	0.030
cg08486397	11	3186792	OSBPL5	TSS1500	0.14	0.16	-0.02	0.041
cg04184683	11	3187446	OSBPL5	TSS1500	0.91	0.88	0.04	0.007
cg09729166	11	3187589	OSBPL5	TSS1500	0.92	0.89	0.03	0.001
cg10891888	12	46765615	SLC38A2	5'UTR	0.05	0.07	-0.01	0.008
cg04838962	12	54765325	ZNF385A	Body	0.94	0.92	0.02	0.010
cg04964471	12	54769641	ZNF385A	Body	0.87	0.84	0.03	0.034
cg01217080	12	54773536	ZNF385A	Body	0.04	0.05	-0.01	0.038
cg05199018	14	101257031	MEG3	5'UTR	0.84	0.87	-0.03	0.020
cg10680608	14	101259648	MEG3	5'UTR	0.86	0.89	-0.03	0.005
cg10515315	14	101290717	MEG3	5'UTR	0.51	0.48	0.03	0.029
cg08698721	14	101294147	MEG3	Body	0.68	0.63	0.05	0.003
cg02546590	14	101295386	MEG3	ExonBnd	0.82	0.86	-0.04	0.009
cg03768174	14	101296630	MEG3	Body	0.80	0.84	-0.04	0.028
cg02412314	14	101296827	MEG3	Body	0.85	0.89	-0.03	0.017

cg05968345	14	101297081	MEG3	Body	0.86	0.89	-0.03	0.001
cg07541415	14	101300082	MEG3	Body	0.84	0.88	-0.03	0.023
cg09971102	14	101309676	MEG3	Body	0.89	0.92	-0.03	0.005
cg00096742	14	101318357	MEG3	Body	0.93	0.92	0.01	0.025
cg07460524	14	101319526	MEG3	Body	0.94	0.95	-0.01	0.004
cg05200614	14	101323508	MEG3	Body	0.92	0.94	-0.02	0.008
cg06163629	20	57414884	GNASAS	Body	0.51	0.48	0.03	0.039
cg10546626	20	57424521	GNASAS	Body	0.72	0.77	-0.05	0.012
cg04457481	20	57426835	GNASAS	TSS1500	0.55	0.50	0.05	0.016
cg03908391	20	57427170	GNASAS	TSS1500	0.63	0.55	0.08	0.005

Table S5. Replication analysis results for genes identified in the methylation studies of the Great Chinese Famine in Qingdao, China

ProbeID	CHR	Position	Gene names	Region	Exposed	Non-Exposed	Difference	*P*
cg04243827	5	3103718	–	–	0.080	0.070	0.010	0.353
cg05083630	9	72169851	APBA1	5'UTR	0.956	0.964	-0.008	0.036
cg06018748	12	59315217	LRIG3	TSS1500	0.883	0.873	0.001	0.456
cg06077226	6	88851970	CNR1	3'UTR	0.952	0.895	0.058	0.152
cg11231670	16	2947301	FLYWCH2	Body	0.846	0.813	0.033	0.083
